# Expression of mammalian GPCRs in *C. elegans *generates novel behavioural responses to human ligands

**DOI:** 10.1186/1741-7007-4-22

**Published:** 2006-07-20

**Authors:** Michelle S Teng, Martijn PJ Dekkers, Bee Ling Ng, Suzanne Rademakers, Gert Jansen, Andrew G Fraser, John McCafferty

**Affiliations:** 1 Wellcome Trust Sanger Institute, Hinxton, Cambridgeshire, UK; 2 MGC Department of Cell Biology and Genetics, Centre for Biomedical Genetics, Erasmus MC, Rotterdam, The Netherlands

## Abstract

**Background:**

G-protein-coupled receptors (GPCRs) play a crucial role in many biological processes and represent a major class of drug targets. However, purification of GPCRs for biochemical study is difficult and current methods of studying receptor-ligand interactions involve *in vitro *systems. *Caenorhabditis elegans *is a soil-dwelling, bacteria-feeding nematode that uses GPCRs expressed in chemosensory neurons to detect bacteria and environmental compounds, making this an ideal system for studying *in vivo *GPCR-ligand interactions. We sought to test this by functionally expressing two medically important mammalian GPCRs, somatostatin receptor 2 (Sstr2) and chemokine receptor 5 (CCR5) in the gustatory neurons of *C. elegans*.

**Results:**

Expression of Sstr2 and CCR5 in gustatory neurons allow *C. elegans *to specifically detect and respond to somatostatin and MIP-1α respectively in a robust avoidance assay. We demonstrate that mammalian heterologous GPCRs can signal via different endogenous G_α _subunits in *C. elegans*, depending on which cells it is expressed in. Furthermore, pre-exposure of GPCR transgenic animals to its ligand leads to receptor desensitisation and behavioural adaptation to subsequent ligand exposure, providing further evidence of integration of the mammalian GPCRs into the *C. elegans *sensory signalling machinery. In structure-function studies using a panel of somatostatin-14 analogues, we identified key residues involved in the interaction of somatostatin-14 with Sstr2.

**Conclusion:**

Our results illustrate a remarkable evolutionary plasticity in interactions between mammalian GPCRs and *C. elegans *signalling machinery, spanning 800 million years of evolution. This *in vivo *system, which imparts novel avoidance behaviour on *C. elegans*, thus provides a simple means of studying and screening interaction of GPCRs with extracellular agonists, antagonists and intracellular binding partners.

## Background

The nematode *C. elegans *represents a simple and experimentally tractable multicellular organism, which has been used to investigate many biological processes, including chemosensory behaviour [1]. It uses only 11 pairs of amphid chemosensory neurons to detect environmental signals. As in mammalian systems, GPCRs play an important role in the detection of sensory signals, and these signals are relayed in the cell by heterotrimeric G proteins. In contrast to mammalian chemosensory systems, the *C. elegans *sensory neurons express multiple GPCRs in each sensory neuron using several Gα subunits per neuron for sensory transduction, thus allowing the nematode to respond specifically to different environmental cues using only a few sensory neurons [2-4].

Current methods utilised to study mammalian GPCR-ligand interactions are mostly *in vitro *systems, which are not always an accurate reflection of *in vivo *interactions. Given that mammalian GPCRs are an important group of drug targets, it would be an advantage to have an accessible *in vivo *system to investigate GPCR interactions with its respective agonists and antagonists. Using *C. elegans *to study *in vivo *GPCR-ligand interactions is an advantage because functional expression of heterologous olfactory receptors in the AWA and AWB olfactory neurons has previously been shown (Milani *et al*) [5] and our unpublished observations. However, olfactory neurons are not directly exposed to the environment, but are embedded in the glial-like amphid sheath cells, making them inaccessible to non-volatile soluble ligands [1]. Critically, they are inaccessible to most non-volatile soluble ligands, precluding the possibility of using such a system for identifying drugs that affect GPCR activity. Hence, we set out to test whether we would elicit ligand-dependent behavioural responses in *C. elegans *by expressing mammalian GPCRs in the ASH and ADL gustatory neurons, as they are directly exposed to the environment allowing access of protein and peptide ligands to the heterologous receptors. In addition, the ASH and ADL neurons express a large variety of Gα subunits [3], increasing the likelihood of GPCR-G_α _protein interaction. The nociceptive neurons, ASH and ADL, drive repulsive responses, [6] and so receptor activation is reflected in an avoidance response on ligand exposure, which can be analysed using robust behavioural assays [7,8]. To test this, we chose to functionally express two medically relevant GPCRs, Sstr2 and CCR5. Somatostatin receptors bind two isoforms of a tetradecapeptide, SST-14 and -28 [9,10]. Both have broad regulatory functions, acting as neurotransmitters in the central and peripheral nervous system and inhibitors of hormone secretion [9,10]. CCR5 is a chemokine receptor that binds MIP-1α (CCL3), MIP-1β (CCL4) and RANTES, and directs chemotactic responses in leucocytes. This receptor is also the route by which HIV-1 infection occurs, making this receptor a therapeutic target in AIDS treatment [11].

Here we show that transgenic *C. elegans *expressing mammalian Sstr2 and CCR5 in the ASH and ADL nociceptive neurons display specific and robust avoidance responses to their respective ligands. The avoidance behaviour to somatostatin in Sstr2 transgenic animals can be inhibited using the somatostatin antagonist cyclosomatostatin. Furthermore, pre-exposure of the GPCR transgenic animals to their respective ligand abolishes this avoidance response without affecting its avoidance behaviour towards other repellent compounds. Heterologously expressed GPCRs are able to signal via different endogenous G_α _subunits depending on which cells they are expressed in, indicating that GPCRs and G_α _proteins are largely conserved in their interaction domains across highly diverged species. Finally, we demonstrate the utility of this avoidance assay by identifying the key residues involved in interaction of somatostatin with its receptor.

## Results

### Transgenic animals expressing heterologous mammalian GPCRs are able to direct specific responses to agonists

**Figure 1:**
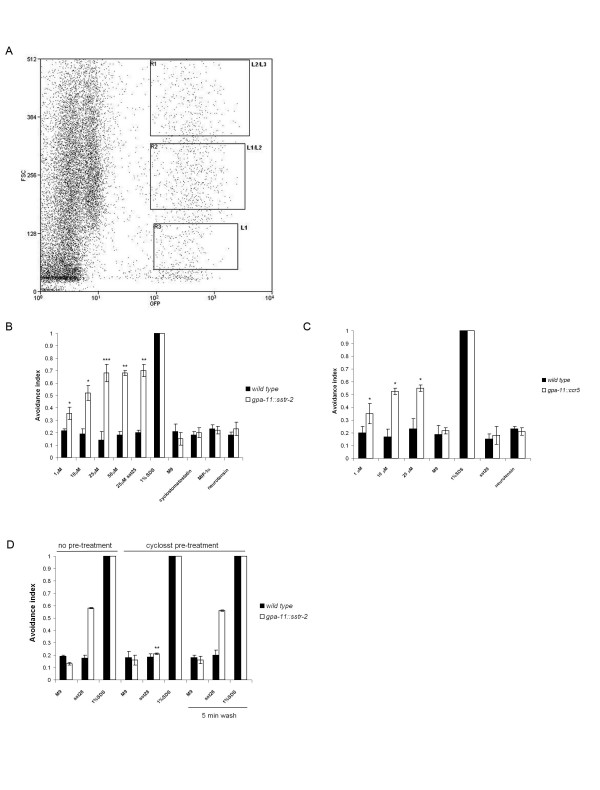
Expression of mouse Sstr2 and human CCR5 in nociceptive neurons in *C. elegans *generates agonist specific avoidance behaviour. (**A**) Flow sorting of transgenic nematodes expressing heterologous GPCRs and gut specific GFP marker (*elt-2::GFP*). Graph shows a plot of forward scatter (size) against GFP fluorescence intensity. GFP-positive animals are sorted into larval stages L1, L2 and L3 according to size with 90% purity and 95% viability (sort gates R1, R2 and R3 respectively). (**B**) Wild type and Sstr2 transgenic animals were tested with 1–50 μM of SST-28. As controls, the responses to 1% SDS, M9 diluent, the unrelated neuropeptides, neurotensin and MIP-1α were determined. Sstr2 transgenic animals did not avoid 25 μM cyclosomatostatin. Both strains display a normal avoidance response to 1% SDS. Asterisks indicate a statistically significant difference between wild type and Sstr2 transgenic animals. (**C**) Avoidance responses of wild type and CCR5 transgenic animals to various concentrations of MIP-1α, M9 diluent, 1% SDS and 25 μM SST-28. Error bars denote the standard error of the mean. Asterisks indicate a statistically significant difference between wild type and CCR5 transgenic animals. (**D**) Animals were either directly tested for their response to M9, SST-28 or 1% SDS or pre-incubated with 25 μM cyclosomatostatin (denoted cyclosst in figure) for 3 minutes before the assays. Pre-treatment with 25 μM cyclosomatostatin abolished the avoidance response of Sstr2 transgenic animals to 25 μM SST-28. Avoidance responses can be recovered by an additional 5 minutes wash to remove cyclosomatostatin. Asterisks indicate a statistically significant difference between cyclosomatostatin treated and untreated transgenic animals. In all panels, each data point represents an average of at least five independent assays for wild-type and each respective strain. Error bars denote the standard error of the mean. (*p ≤ 0.05, **p ≤ 0.01, ***p ≤ 0.005). Avoidance index = number of worms behind barrier/total number of worms

To express mammalian GPCRs in the ASH and ADL nociceptive neurons, we cloned the receptor genes downstream of the *gpa-11 *promoter, which drives expression in these neuron pairs [3]. Following injection, transgenic animals were visualised using a gut-expressed *elt-2::GFP *construct, allowing selection of live transgenic animals from mixed populations using a flow sorter (MoFlo) modified with a 150-μm flow-cell tip (Figure 1A; see Additional file 1 and 2).

Sorted populations of Sstr2 and CCR5 transgenic animals (denoted as *gpa-11::sstr2 *and *gpa-11:ccr5 *respectively in figures) were tested for their response to the native ligands using a soluble compound avoidance assay adapted from Wicks *et al *[7]; (see Materials and methods). Wild type and Sstr2 transgenic animals were tested for their response to varying concentrations of SST-28. In contrast to wild-type animals, Sstr2 transgenic animals exhibited avoidance of SST-28 (Figure 1B). The strongest response was observed with 25 μM SST-28. Ovine isolated SST-25 and human SST-28 have previously been found to have higher biological activity than SST-14 [12]. The behaviour of the Sstr2 transgenic animals is consistent with this; SST-25 and SST-28 induced stronger avoidance responses than SST-14 (Figures 1B, 4). As a control, we tested the response of this strain to neurotensin and MIP-1α. Neither compounds elicited a response in the Sstr2 transgenic animals, confirming specificity of GPCR-ligand interaction (Figure 1B).

**Figure 2:**
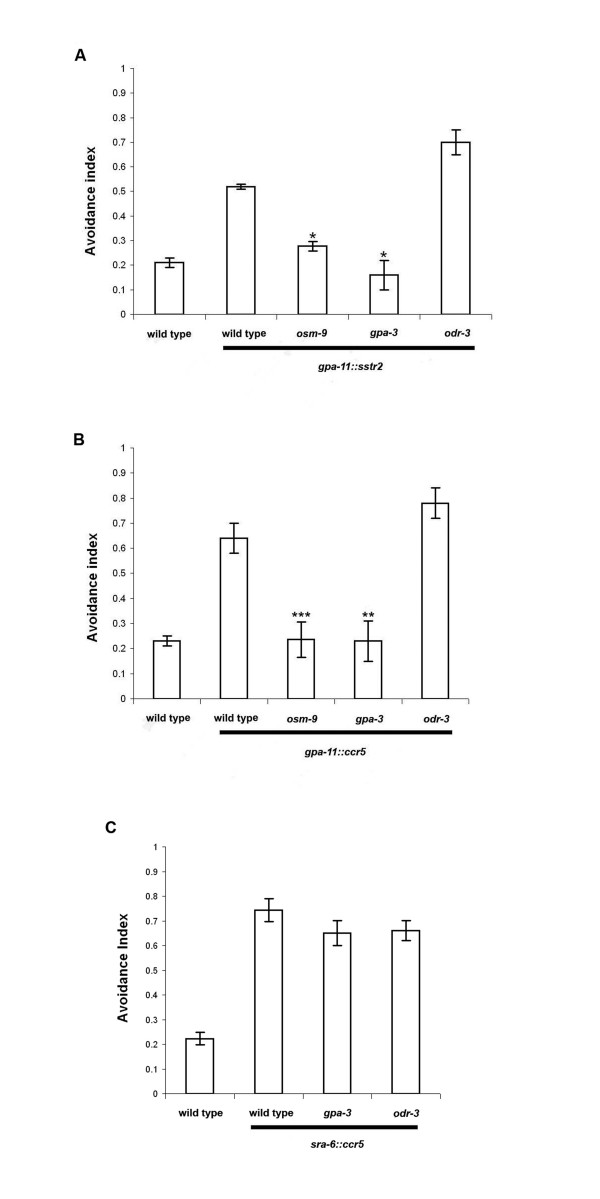
Heterologously expressed GPCRs are integrated into an endogenous signalling pathway in *C. elegans*. Mutation of the TRPV channel subunit *osm-9 *and the Gα subunit *gpa-3 *fully abolished avoidance of (**A**) 25 μM SST-28 in Sstr2 transgenic animals and (**B**) 25 μM MIP-1α in CCR5 transgenic animals. Mutation of Gα subunit *odr-3 *did not affect these responses. (**C**) In contrast, mutation of either *gpa-3 *or *odr-3 *did not have a significant effect on avoidance response in *sra6::CCR5 *transgenic animals. Each data point represents an average of at least 3 independent assays for wild type and each respective strain. Error bars denote the standard error of the mean. Asterisks indicate a statistically significant difference between *osm-9 *or *gpa-3 *mutant animals and wild type animals carrying the Sstr2 or CCR5 transgenes (*p ≤ 0.05, **p ≤ 0.01, ***p ≤ 0.005).

In order to confirm that other GPCRs could be expressed functionally in *C. elegans*, we generated transgenic animals that express CCR5 in the ASH/ADL neurons. Transgenic strains were tested for avoidance of MIP-1α, SST-28 and neurotensin. CCR5 transgenic animals only showed avoidance of MIP-1α, not of the control peptides (Figure 1C). An optimal avoidance response was obtained with 10 μM MIP-1α. In addition, we also expressed CCR5 in the ASH neurons using the *sra-6 *promoter, which also gave a robust avoidance response to 25 μM MIP-1α(Figure 2C). This demonstrates that mammalian GPCR expression in the ASH neurons is sufficient to generate avoidance behaviour to its ligands. We also tested the response of the Sstr2 and CCR5 transgenic animals to SST-28 and MIP-1α using the dry-drop test [8]. The average frequency of avoidance for each tested compound in this single animal assay was very similar to that in the population assay (Additional file 3).

The ASH and ADL neurons of *C. elegans *normally direct avoidance responses to environmental agents such as low pH and sodium dodecyl sulphate (SDS) [6]. When exposed to a barrier of 1% SDS, neither transgenic strain crossed the barrier, suggesting that normal avoidance behaviour is unaffected by expression of heterologous GPCRs. We have thus shown that expression of mammalian GPCRs in transgenic animals generates a specific avoidance response towards its respective agonists.

### Sstr2 antagonist, cyclosomatostatin can inhibit response of Sstr2 transgenic animals to somatostatin

To analyse the specificity of ligand-receptor interaction reflected in the avoidance response, Sstr2 transgenic animals were tested with a competitive somatostatin antagonist, cyclosomatostatin [cyclo(D-Trp-Lys-Thr-Ahep-Phe)], which blocks somatostatin binding to its receptors [13]. Pre-exposure of Sstr2 transgenic animals to 10 μM of cyclosomatostatin in liquid for 3 minutes eliminated most of the avoidance behaviour towards 25 μM SST-28 (Figure 1D). Higher concentrations of cyclosomatostatin and longer incubation times tend to cause paralysis in both wild-type and transgenic animals (results not shown). We also tested for reversibility of this antagonistic effect by allowing the worms to recover for 5 minutes by washing them after the 3 minute pre-incubation step. The resulting effect was a recovery of the Sstr2 transgenic animals' avoidance behaviour to SST-28 (Figure 1D). Hence, the avoidance response to SST-28 could be restored in 5 minutes after antagonist pre-exposure.

### Mammalian heterologous GPCRs can signal via different endogenous G_α _subunits in *C. elegans*

Our results confirm that the biological properties of the GPCRs can be translated into the altered sensory behaviour of the transgenic animals, suggesting that heterologous GPCRs use *C. elegans *signal transduction in nociceptive neurons. *gpa-11 *is expressed predominantly in ADL neurons, and to a lesser extent in ASH neurons [3]. The ASH and ADL neurons express a variety of G_α _proteins. Little is known about the molecules used in the ADL cells. Nociceptive signalling in ASH involve the G_α _proteins ODR-3 [14] and GPA-3 [15]. These in turn lead to downstream activation of TRPV calcium-channel OSM-9 or OCR-2 [16,17]. To identify some of the molecules involved, we tested several mutants known to affect avoidance behaviour. Inactivation of *osm-9 *or *gpa-3 *fully abolished the response to SST-28 and MIP-1α in Sstr2 and CCR5 transgenic animals respectively, whereas inactivation of *odr-3 *did not (Figure 2A and 2B). These results indicate that the heterologous GPCRs, expressed predominantly in the ADL signals via GPA-3 to activate OSM-9. In contrast, the avoidance response to MIP-1α of *sra-6::ccr5 *animals expressing CCR5 predominantly in the ASH neurons (with a low level in ASI) were not affected by removal of *gpa-3 *or *odr-3 *(Figure 2C) [18]. Given that *sra-6 *is weakly expressed in ASI that mediates attraction behaviour, this may have a subtle effect on avoidance mediated by the ASI neurons, although is unlikely to affect the avoidance behaviour of the transgenic animals towards its ligands. Our results indicate that CCR5 can activate different *C. elegans *G_α _subunits, depending in which cells it is expressed in. In ASH neurons, CCR5 could signal redundantly via GPA-3 or ODR-3 or any of the other G_α _subunits present in these neurons. These results illustrate a level of promiscuity in the interactions between heterologous GPCRs and the endogenous signalling components of *C. elegans*.

### Pre-exposure to native agonists causes receptor desensitisation and adaptation behaviour in Sstr2 and CCR5 transgenic animals

**Figure 3:**
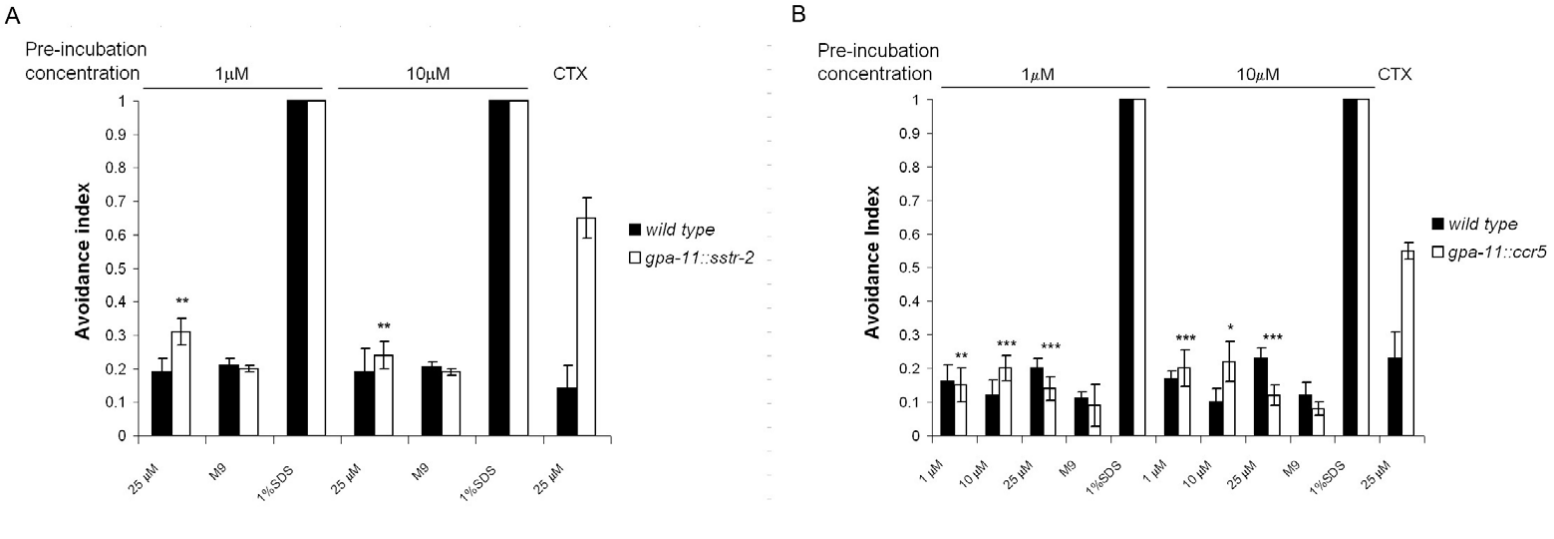
Sstr2 and CCR5 transgenic animals mutants are desensitised by pre-exposure to agonists. (**A**) Pre-exposure of Sstr2 transgenic animals to 1 μM and 10 μM SST-28 prior to the assay strongly reduced or even abolished avoidance behaviour. (B)Pre-exposure of CCR5 transgenic animals to 1 μM and 10 μM MIP-1α fully abolished avoidance of 1 μM, 10 μM and 25 μM MIP-1α. In both cases, pre-exposure to the agonist did not affect the responses towards other repellents. Animals were washed in buffer as control. For each panel, each data point represents an average of at least five independent assays for wild type and each respective strain. Error bars denote the standard error of the mean. Asterisks indicate a statistically significant difference between transgenic animals pre-exposed to agonist or only to chemotaxis (CTX) buffer (*p ≤ 0.05, **p ≤ 0.01, ***p ≤ 0.005).

Activation of GPCRs in both worms and mammals eventually leads to receptor desensitisation through phosphorylation by GPCR kinases (GRKs) and arrestins [19-21]. To study whether the response of the Sstr2 and CCR5 transgenic animals to their respective ligands was subject to desensitisation (leading to adaptation), the animals were pre-exposed to either 1 μM or 10 μM of the respective peptides in liquid for 10 minutes in liquid before testing the avoidance response. The avoidance behaviour of Sstr2 transgenic animals in response to 25 μM SST-28 was strongly reduced after pre-exposure to 1 μM SST-28, whereas pre-exposure to 10 μM SST-28 could fully abolish avoidance of SST-28 (Figure 3A). The avoidance behaviour to other repellents such as 1% SDS was not affected by the pre-exposure to SST-28, suggesting that SST-28 adaptation is independent from the pathway that signals avoidance of 1% SDS. Similarly, CCR5 transgenic animals were pre-exposed to 1 μM and 10 μM of MIP-1α and tested at three different MIP-1α concentrations. Full adaptation to MIP-1α was achieved with both pre-exposure concentrations; however, pre-exposure did not affect other avoidance responses (Figure 3B). These results demonstrate that avoidance of SST-28 and MIP-1α mediated by their respective mammalian GPCRs in *C. elegans *is subject to specific adaptation. We propose that the *C. elegans *signalling components involved in receptor desensitisation interact with the heterologous GPCRs.

### Trp8 and Lys9 of SST-14 are essential for Sstr2 activation

**Figure 4:**
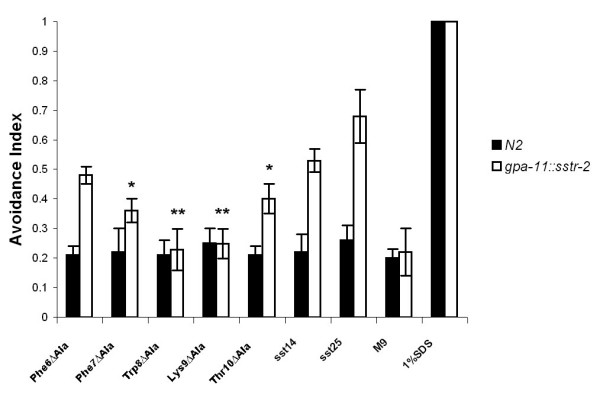
Identification of SST-14 residues involved in Sstr2 activation. Sstr-2 transgenic animals were tested for response to SST-14 and five variants with single alanine substitutions at residues 6–10. No avoidance behaviour was observed with 25 μM of Trp8Δ Ala and Lys9Δ Ala SST-14 analogues while residual avoidance behaviour was found with Phe7Δ Ala and Thr10Δ Ala. Sstr2 transgenic animals were repelled by the Phe6Δ Ala analogue, indicating that Phe6 is not essential for receptor:ligand interaction. Each data point represents an average of at least five independent assays for wild type and each respective strain. Error bars denote the standard error of the mean. Asterisks indicate a statistically significant difference between the avoidance index of the transgenic animals for the Trp8Δ Ala and Lys9Δ Ala analogues and native SST-14 agonist (*p ≤ 0.05, **p ≤ 0.01).

Given that heterologous GPCRs can be expressed functionally in nociceptive neurons, we sought to investigate the structure-function relationship by screening SST-14 analogues. Structure activity studies on SST-14 have suggested that the amino-acid residues Phe7, Trp8, Lys9 and Thr10 are important in receptor-ligand interaction, with Trp8 and Lys9 being essential, whereas Phe7 and Thr10 can undergo minor conserved substitutions [22]. We investigated the role of these residues by testing the five peptides analogues, where residues 6–10 of SST-14 are individually substituted with Ala. The Sstr2 transgenic animals showed a completely attenuated response towards the Trp8Δ Ala and Lys9Δ Ala analogues, whereas partial avoidance responses were observed to both Phe7Δ Ala and Thr10Δ Ala (Figure 4). No significant difference in avoidance response was observed with Phe6Δ Ala, indicating that this residue is not essential for receptor-ligand interaction.

## Discussion

G protein coupled receptors (GPCRs) represent the largest and most diverse family of proteins in the human genome [23]. Given that this family of proteins play key roles in many biological processes, they constitute one of the principal targets for drug development. The *C. elegans *sensory system is exceptionally well suited for heterologous GPCR expression given that each neuron expresses multiple endogenous GPCRs [2]. Furthermore, multiple G_α _subunits are expressed in each chemosensory neuron [3] allowing cross-talk between a single GPCR and potentially multiple G_α _proteins. Moreover, the gustatory neurons are exposed to the external environment, making it accessible to water-soluble ligands. Here we show that expression of heterologous GPCRs in gustatory neurons under the control of two different promoters, *gpa-11 *(ADL and ASH expression) and *sra-6 *(ASH expression with weak expression in ASI), can drive repulsive responses to the respective ligands presented in soluble pure form. The behavioural response to the ligands can be tested using robust avoidance assays, making this an ideal *in vivo *system to study receptor-ligand interactions. Sstr2 and CCR5 transgenic animals were able to specifically avoid the respective agonists without affecting avoidance behaviour towards other repellents. Similar responses were observed in multiple transgenic strains with different promoters using both the population and single-animal avoidance assays. The fidelity of the system extends to antagonists, as pre-exposure of Sstr2 transgenic animals to a somatostatin antagonist, cyclosomatostatin, inhibited avoidance response to somatostatin. The antagonistic effect was reversible given that the avoidance behaviour could be recovered by washing the transgenic animals. This provides further evidence for specific receptor-ligand interaction.

It has previously been shown that widespread promiscuity occurs in the interactions of mammalian GPCRs with mammalian G_α _proteins [23,24]. Our results demonstrate that mammalian GPCRs integrate into the normal endogenous G_α _protein signalling pathway in the worm, showing a remarkable evolutionary plasticity in these interactions, spanning 800 million years [25]. Rat Sstr2 has previously been demonstrated to interact with endogenous yeast G_α _protein, Gpa1p, when the receptor was expressed in *Sacchromyces cerevisiae*, which provides further evidence that G proteins across highly diverged species are largely conserved in their interaction domains [26]. Moreover, functional expression of mammalian bitter-taste receptors (of the T2R family) and TRPV (VR1) channels in ASI and ASH neurons have been reported, supporting the suitability of *C. elegans *as a heterologous expression system [17,27]. In this paper, we show that several G_α _proteins are involved in downstream signalling in the ASH neurons. Given the abundance of G_α _proteins in these neurons, it would be interesting to examine the individual roles of G_α _proteins involved in signalling with heterologous GPCRs.

Attenuation of GPCR signalling by GRKs and arrestins is important for an organism to maintain a homeostatic cellular environment [19-21]. Desensitisation also ensures that receptors are ready for the next coming wave of input stimuli. We found that pre-exposure to varying concentrations of the ligand leads to adaptation of the response to the ligand but not to other repellents. This suggests that the heterologously expressed mammalian GPCRs in *C. elegans *are subjected to desensitisation by the endogenous machinery, further supporting the notion that heterologous GPCRs are integrated into the endogenous signalling pathway.

The system for heterologous expression of functional GPCRs in *C. elegans *described here provides a novel means of screening for agonists and antagonists, and for carrying out structure function studies on GPCRs and their ligands. To illustrate this potential, we measured receptor activation on a series of SST-14 mutant peptides, in which amino acids at positions 6–10 were individually changed to alanine. The avoidance response to Trp8Δ Ala and Lys9Δ Ala SST-14 analogues was abolished while reduced avoidance behaviour was found with Phe7Δ Ala and Thr10Δ Ala. Sstr2 transgenic animals were repelled by the Phe6Δ Ala analogue, indicating that Phe6 is not essential for receptor-ligand interaction. This work confirmed and extended the previous finding, based on activity of hexapeptide analogues, that residues 7–10 contain key elements necessary for its biological activity of somatostatin [13]. This illustrates the practical utility of this system for identifying structure-function relationships and for screening soluble compounds by measuring behavioural responses in GPCR transgenic animals.

## Conclusion

Our work describes the generation of novel behavioural response in *C. elegans *to exogenous human ligands. This provides a powerful platform for exploring GPCR-ligand interactions in an animal model *in vivo*. Furthermore, the 'synthetic' response holds potential in basic studies of behaviour and higher learning in *C. elegans*. By exploiting the gustatory chemosensory system in *C. elegans*, we have developed a novel and rapid assay to study interaction of medically important receptors with both extracellular and intracellular partners and drug candidates.

## Methods

### Strains and plasmids

Nematodes were grown at 16°C or 20°C on *E. Coli *strain OP50 using standard methods [28]. Wild-type animals were *C. elegans *variety Bristol strain N2. Strains used in this study are *odr-3*(*n1605*), *gpa-3*(*pk35*) and *osm-9(ky10)*.

### Reagents

SST-14, -28, -25 and its analogues were purchased from Bachem, UK. MIP-1α was obtained from GeneFlow, UK. Peptides were dissolved in M9.

### Molecular biology

Full-length mouse Sstr2 and human CCR5 were cloned downstream of a 1.5 kb fragment of the *gpa-11 *promoter into pUC119. The 3' UTR of *unc-54 *was cloned downstream of both genes. The fragment containing CCR5 and the *unc-54 *3'UTR was subcloned into an *sra-6*::GFP construct.

### Transgenic strains

Germ line transformation was carried out as described by Mello *et al. *[29] using 40 ng/μl of *gpa-11::sstr2*, *gpa-11::ccr5 *with 100 ng/μl of *elt-2:gfp *plasmid, or 100 ng/μl of *sra-6::ccr5 *with 25 ng/μl of *elt-2:gfp *[30]. As the transgenic animals carry non-integrated arrays that could cause a degree of fluctuation in the assay, at least three transgenic strains were tested before picking out the best responding strain for repeating assays. For the mutant analysis, the best responding non-integrated strain was crossed with *osm-9*(*ky10*), *gpa-3*(*pk35*), *odr-3*(*n1605*) mutant. The mutant alleles were traced by PCR. At least three transgenic strains were tested before picking out the best responding strain for repeating assays.

### FACS sorting conditions

Animals were washed with M9 and filtered through a 100-μm mesh filter. Appropriate sorting of each developmental stage was verified using epifluorescence microscopy. The animals were then grown for a day prior to testing in a soluble compound avoidance assay (see Additional file 1 for more details).

### Soluble compound avoidance assay

Mutant avoidance behaviour was assessed on rectangular four-well plates (Nunclon, UK) using an assay adapted from Wicks *et al. *[7]. This reduces the amount of avoidance compound used. Each compartment was filled with 12 ml CTX agar (2 % agar, 5 mM potassium phosphate buffer (pH 6.6), 1 mM CaCl_2, _1 mM MgSO_4_). A thin line of the soluble ligand was applied across one end of the well about 25 mm from the end of the plate (note that the ligand concentrations given here are the applied concentrations for the assay). Adult animals were washed three times in CTX buffer (5 mM potassium phosphate buffer (pH 6.6), 1 mM CaCl_2, _1 mM MgSO_4_). When the test compound was absorbed into the agar, about 50 animals were placed 10 mm behind the line of the compound in about 5 μl of CTX buffer. A volatile attractant, 1:10 benzaldehyde, was added to the opposite end of the plate. The avoidance index was calculated by counting the number of worms that did not crawl past the boundary after 30 minutes, divided by the total number of worms applied. The assay was performed for 30 minutes as the avoidance index did not change significantly after 30–50 minutes into the assay. Avoidance index = number of worms behind barrier/total number of worms

### Statistical analysis

Statistical analysis of behavioural data was performed using the paired Student's *t*-test. If multiple groups were tested, statistical significance was determined by ANOVA. An α level of 0.05 was used in all tests. All results are given as mean ± SEM

## Authors' contributions

The transgenic animals were generated by MST and MPJD. Experiments were carried out mainly by MST with assistance from GJ, MPJD and SR. MoFlo analysis was performed by BLN. Experimental design was performed by MST, GJ and JM. GJ, AF and JM were involved in revising the manuscript critically for important intellectual content. All authors read and approved the final manuscript.

## Supplementary Material

Additional File 1 Teng *et. al. *34 kb Method for flow sorting and figure legend for additional file 2. Click here for file

Additional File 2 Teng *et. al. *4.2 MB Flow sorting table and figure. Summary table for MoFlo sorting conditions and confocal images of sorted transgenic populations Click here for file

Additional File 3 Teng *et. al. *25 kb Comparison of avoidance indices of population vs single animal avoidance assay (dry drop test) Click here for file
