# Supplementary material for: Expression of mammalian GPCRs in C. elegans generates novel behavioural responses to human ligands
Source: BMC Biol. 2006 Jul 20;4:22. doi: 10.1186/1741-7007-4-22 (PMC1550261; doi:10.1186/1741-7007-4-22)
Supplement: Additional File 1 — Teng et. al. 34 kb Method for flow sorting and figure legend for additional file 2. [file 1741-7007-4-22-S1.doc]

**Additional information 1**

**Methods**

**FACS/MoFlo worm sorting**

All experiments were carried out using a Dako Cytomation four-way cell sorter (MoFlo®), equipped with a Coherent Sapphire^TM^ 488-nm argon ion laser in the primary position (first laser as trigger). Flow sorting was carried out using Cytomation Sort Unit (CSU). The degree of amplitude and frequency of the drive was optimised by drop-delay test using 10 μm fluorescence beads (Flow Check, Beckman Coulter Inc.). All worm samples were filtered through 150-μm mesh filters (Celltrics^TM^ 150 μm; Partec GmbH) before FACS sorting. The GFP transgenic nematodes were sorted either directly onto agar plates or into 1-ml eppendorf tubes containing 100 μl of M9 buffer. The sorted fractions were then centrifuged briefly at 200 g for 1 minute at 4 °C before microscopy analysis to reduce larvae movement during photograph capture. The nematode samples were analysed at an approximate rate of 100 worms/minute. A predetermined number of sort events of *elt-2:gfp* transgenic animals were sorted directly onto agar plates. The viability of sorted worms was determined by counting the number of live worms using fluorescence microscopy or by counting the number of live worms after 1 hour's incubation at 20 °C. Viability of 95% was achieved for each sorting process. The purity of sorting was verified by examining the sorted fractions using confocal microscopy (Biorad Radiance 2100; Carl Zeiss Cell Science Ltd, Germany). Images acquired from confocal microscopy were recorded using LaserSharp 2000 software. Mixed populations of *Caenorhabditis elegans* containing between 3% and 5% GFP-positive populations were selected on three different gates set based on their size. The purity of the sorted fractions was determined by counting the number of GFP-transgenic nematodes from the total sorted fraction. Purity of 90–100% was achieved using the optimised sort conditions for *C. elegans* (Table 1).

**Figure 1** Sorting *C. elegans* by flow cytometry**.** The isolation of transgenic progeny prior to setting up assays requires laborious separation from the non-expressors by picking individual worms. Sorting worms by MoFlo allows the rapid isolation of transgenic worms and is easily reproducible. The use of conventional flow cytometry allow the rapid enrichment and separation of a heterogeneous transgenic population (L1 to L3) from the non-expressors. The separation is based on animal size and GFP fluorescence. The separated populations have 90-100% viability with 95% purity.

**Table 1** summarises the optimised conditions required for effective flow sorting. A sorting rate of approximately two worms/second was achieved based on a population containing 3% GFP-positive worms. The percentage of sorted events varied according to the proportion of transgenic animals in the sample. **A:** A mixed heterogeneous population of *elt-2::GFP* and non *elt-2::GFP* expressing animals. Sorted populations were analysed by bright-field and confocal fluorescence microscopy into three distinct fractions of (**b)** L1; (**c)** L1/L2 and (**d)** L2/3.
