# Supplementary figures and images for: Expression of mammalian GPCRs in C. elegans generates novel behavioural responses to human ligands
Source: BMC Biol. 2006 Jul 20;4:22. doi: 10.1186/1741-7007-4-22 (PMC1550261; doi:10.1186/1741-7007-4-22)

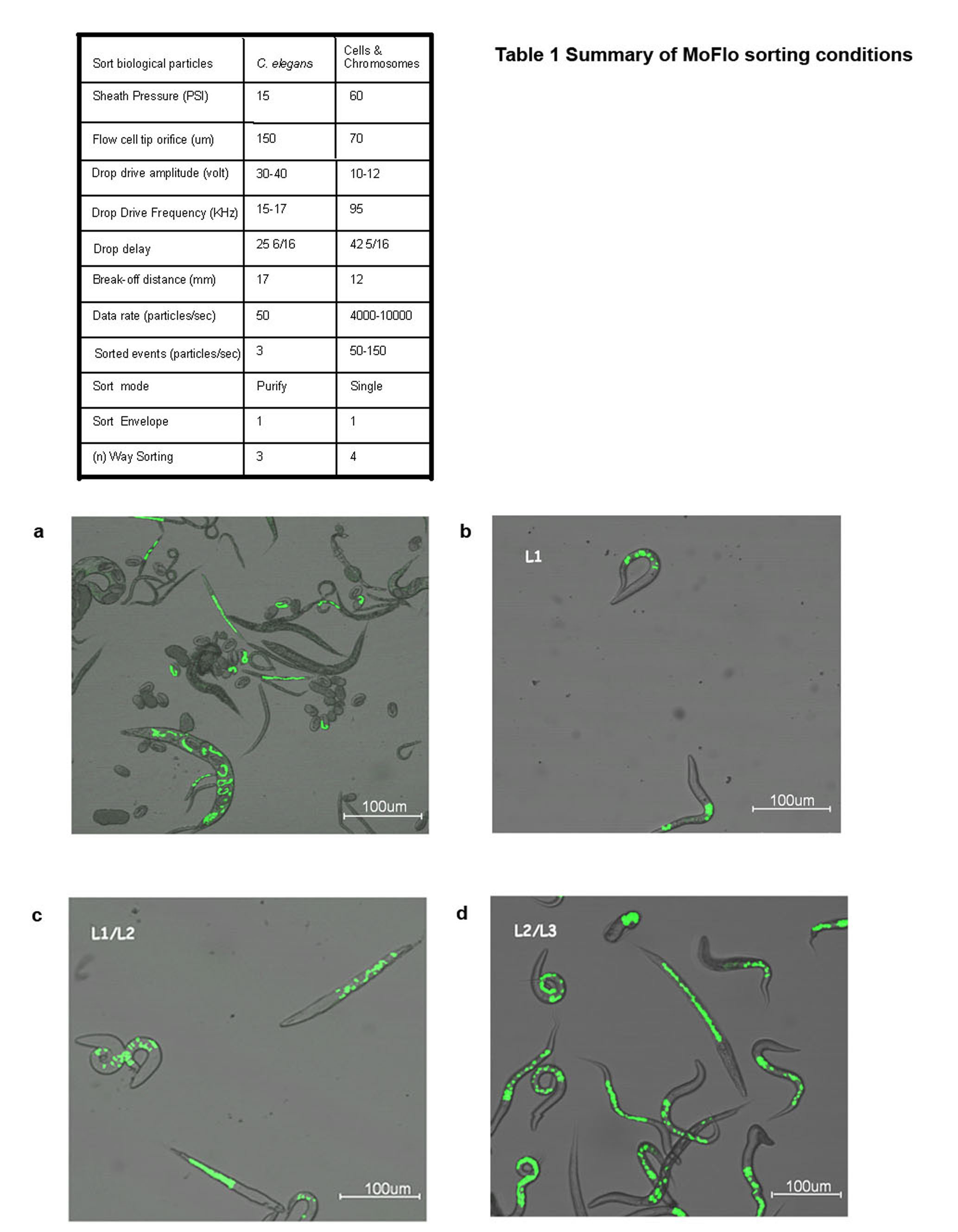

Supplement: Additional File 2 — Teng et. al. 4.2 MB Flow sorting table and figure. Summary table for MoFlo sorting conditions and confocal images of sorted transgenic populations [file 1741-7007-4-22-S2.jpeg]

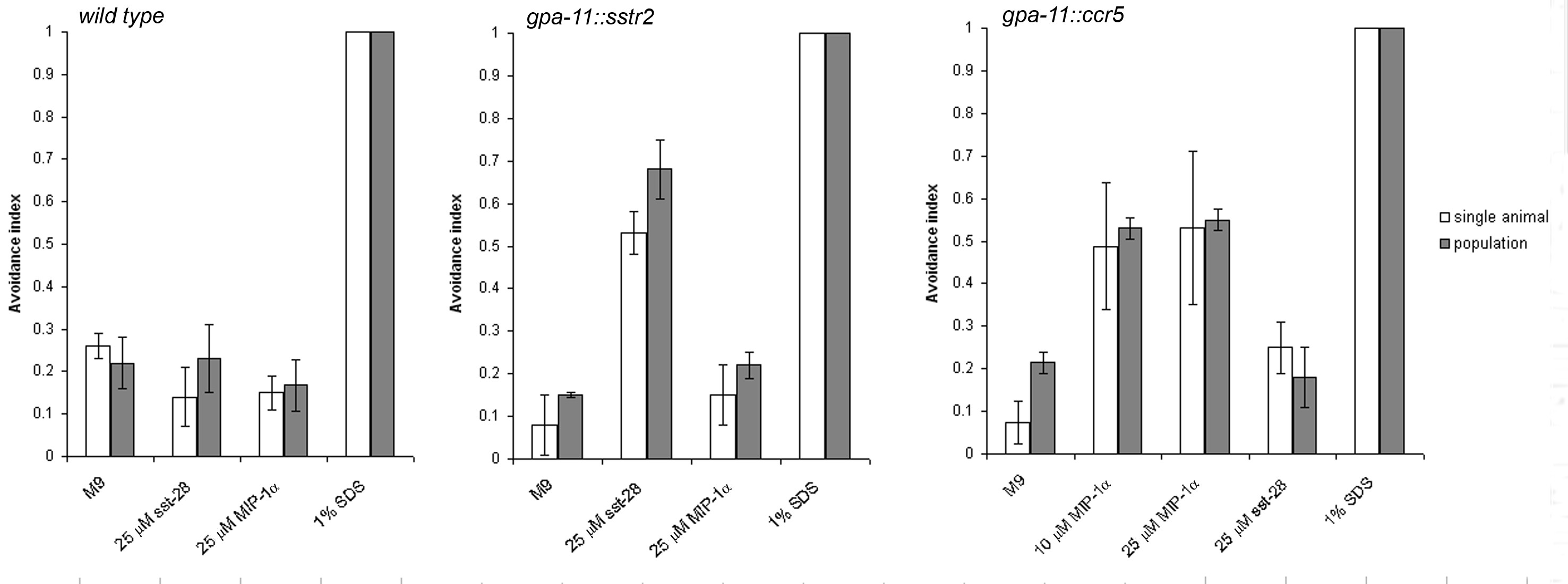

Supplement: Additional File 3 — Teng et. al. 25 kb Comparison of avoidance indices of population vs single animal avoidance assay (dry drop test) [file 1741-7007-4-22-S3.jpeg]
